# Diaphragmatic rupture secondary to trauma from falling sacks: A case report

**DOI:** 10.1002/ccr3.7427

**Published:** 2023-05-28

**Authors:** Oshan Shrestha, Sunil Basukala, Sagun Karki, Niranjan Thapa, Niraj Joshi, Lochan Shrestha, Melina Shrestha

**Affiliations:** ^1^ College of Medicine Nepalese Army Institute of Health Sciences Kathmandu Nepal; ^2^ Department of Surgery Nepalese Army Institute of Health Sciences Kathmandu Nepal

**Keywords:** diaphragmatic hernia, mesh repair, shortness of breath, trauma

## Abstract

**Key Clinical Message:**

Diaphragmatic hernia does not only occur during high velocity impact or penetrating injury, but also can occur when heavy loads impact the torso. Diaphragmatic hernia must be ruled out in a patient with polytrauma with a chest X‐ray at the least.

**Abstract:**

Trauma‐induced diaphragmatic hernia is a protrusion of abdominal contents through the defect in diaphragm and is an uncommon and less heard of injury. This case report conveys that diaphragmatic hernia should be ruled out in any polytrauma case presenting with shortness of breath with the chest X‐ray at the least.

## Graphical abstract

High left hemidiaphragm following diaphragmatic rupture secondary to trauma.

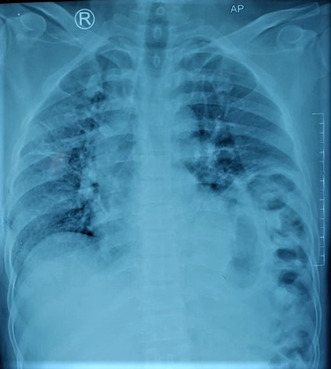

## 1 INTRODUCTION

Trauma‐induced diaphragmatic hernia, protrusion of abdominal contents through the defect in diaphragm, has reported incidence of 0.8%–8%. This is an uncommon injury resulting from high velocity impact or penetrating injury to the torso.^1^, ^2^ Diaphragmatic hernia may remain silent or may have acute presentation such as marked respiratory distress or it may manifest late with sequelae of herniation.^3^, ^4^ Left sided diaphragmatic injury and hernia are more frequently encountered compared to right sided injury and hernia while bilateral injury is much more uncommon.^5^ There exist a positive gradient of 7–20 cm of H_2_O between intraperitoneal and intrapleural pressures. During the impact, this gradient exceeds 100 cm of H_2_O and pose risk of diaphragmatic injury. Due to continuous diaphragmatic motion, the defect cannot heal and instead results in progression of the size of the defect.^6^ In this case report, we present a case of a 58‐year‐old laborer who developed left sided diaphragmatic hernia after sustaining impact over his torso from multiple rice‐sacks falling from a height of approximately 3 m.

This case report is presented by following CARE guidelines.^7^

## 2 CASE REPORT

A 58‐year‐old man, a laborer by profession, was presented to the emergency of our tertiary care center with issues of shortness of breath and lower back pain that had started 6 h before the presentation. Otherwise a healthy individual with no past medical/surgical history, the patient sustained injury over his torso after multiple sacks of rice fell over him from a height of approximately 3 m. The shortness of breath and lower back pain had started immediately after the impact. There was no history of loss of consciousness, vomiting, abnormal body movements, bleeding from orifices or any external wounds, and bruises after the event.

### 2.1 Timeline

**FIGURE 1:**
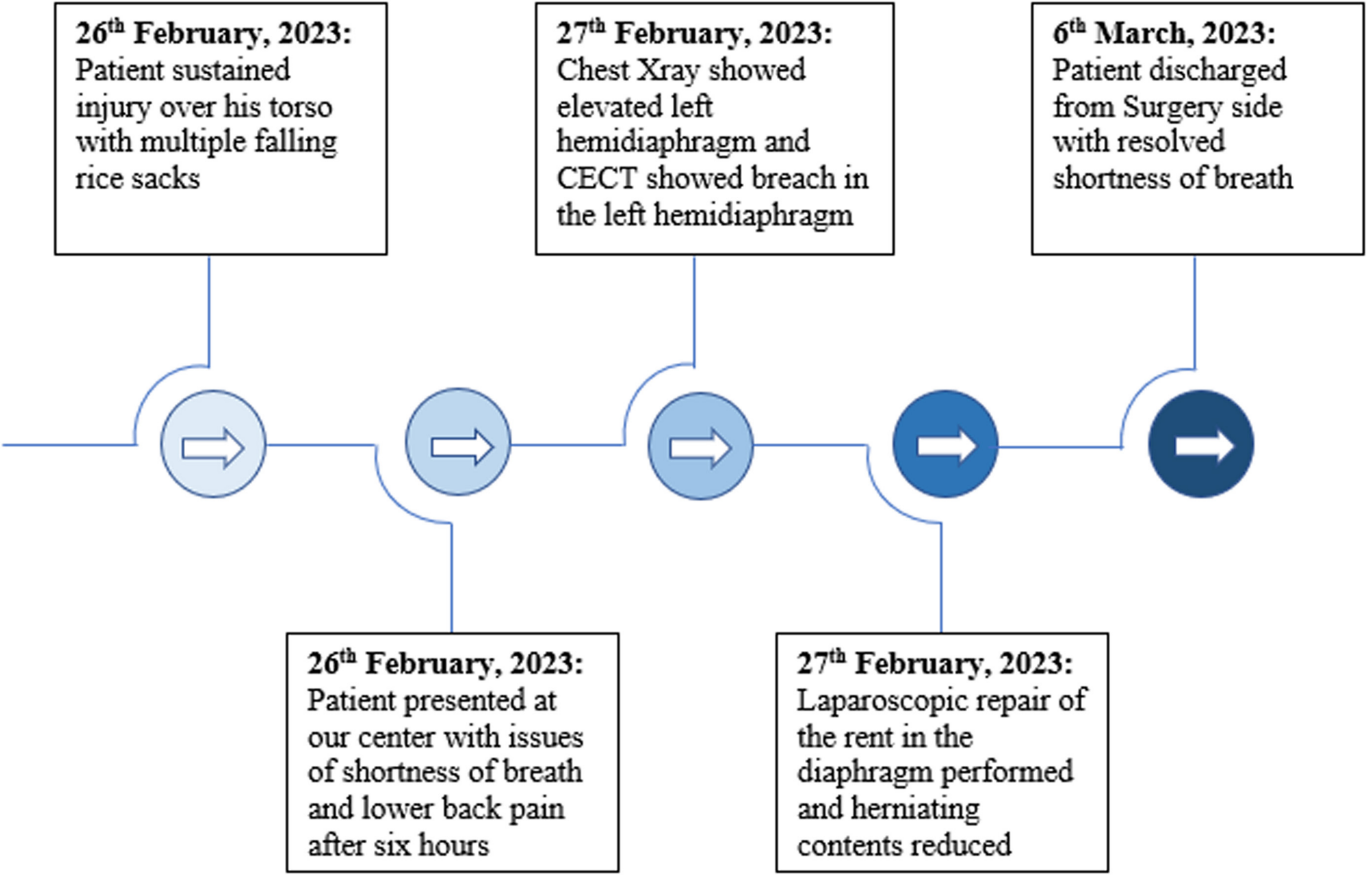
Timeline showing major events.

Patient sustained injury over his torso from multiple rice‐sack falling from the height of approximately 3 m. Patient developed defect over left hemidiaphragm through which abdominal contents herniated. Surgical repair of the defect was performed. Figure 1 shows timeline of major events.

### 2.2 Diagnostic assessments

Physical examination at the time of presentation showed 15 Glasgow Coma Scale (GCS) score, blood pressure of 140/90 mm of mercury, pulse rate of 94 beats per minute, respiratory rate of 26 breaths per minute, oxygen saturation at room air of 93%, and the body temperature was 98°F. Respiratory examination revealed dull sound on percussion, and decreased breath sounds and bowel sounds on auscultation over the left chest region. Abdomen of the patient was scaphoid shaped, and tenderness was present over left upper quadrant. Patient also had mild swelling and tenderness over the lumbar vertebrae (LV) region. Neurological examination showed 0/5 motor score and 1/2 sensory score over bilateral lower limb for L_4,_ L_5_, and S_1_ levels. Other examination findings were normal, and all the baseline laboratory workups were also normal.

**FIGURE 2:**
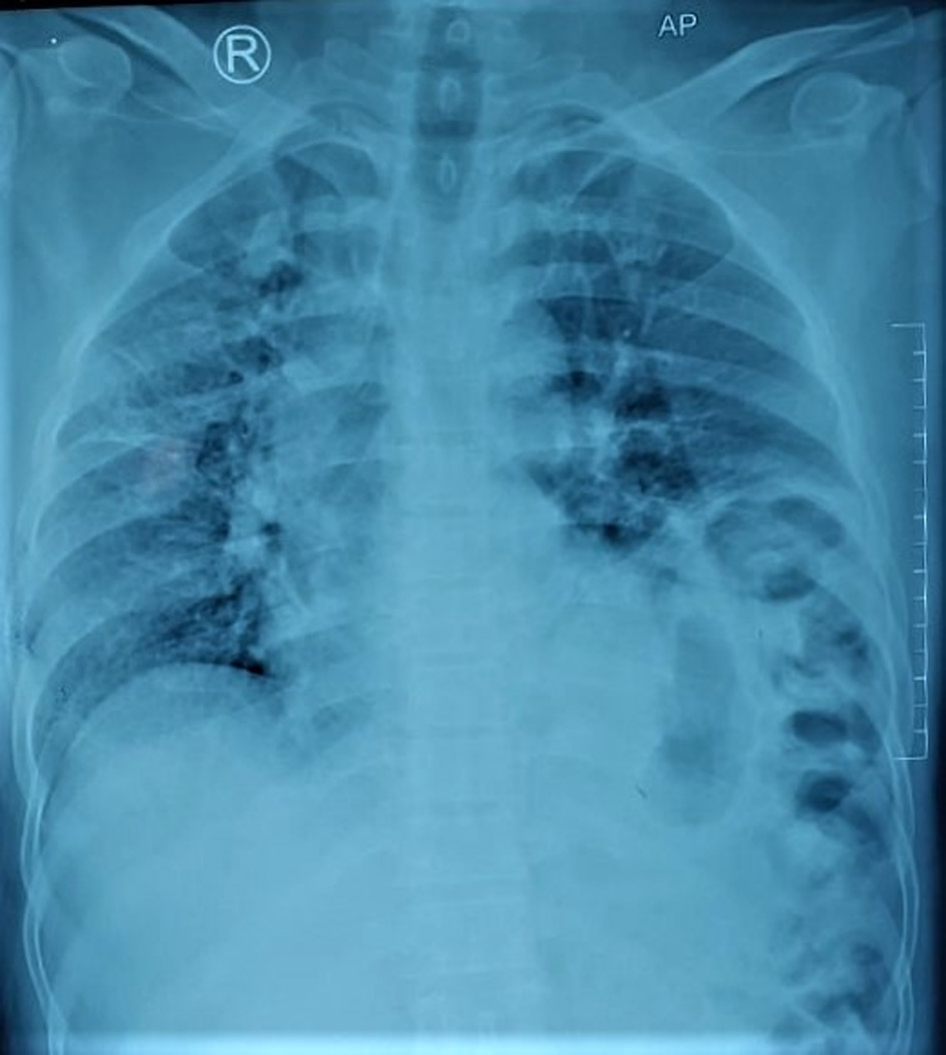
Chest x‐ray showing elevated left hemidiaphragm.

**FIGURE 3:**
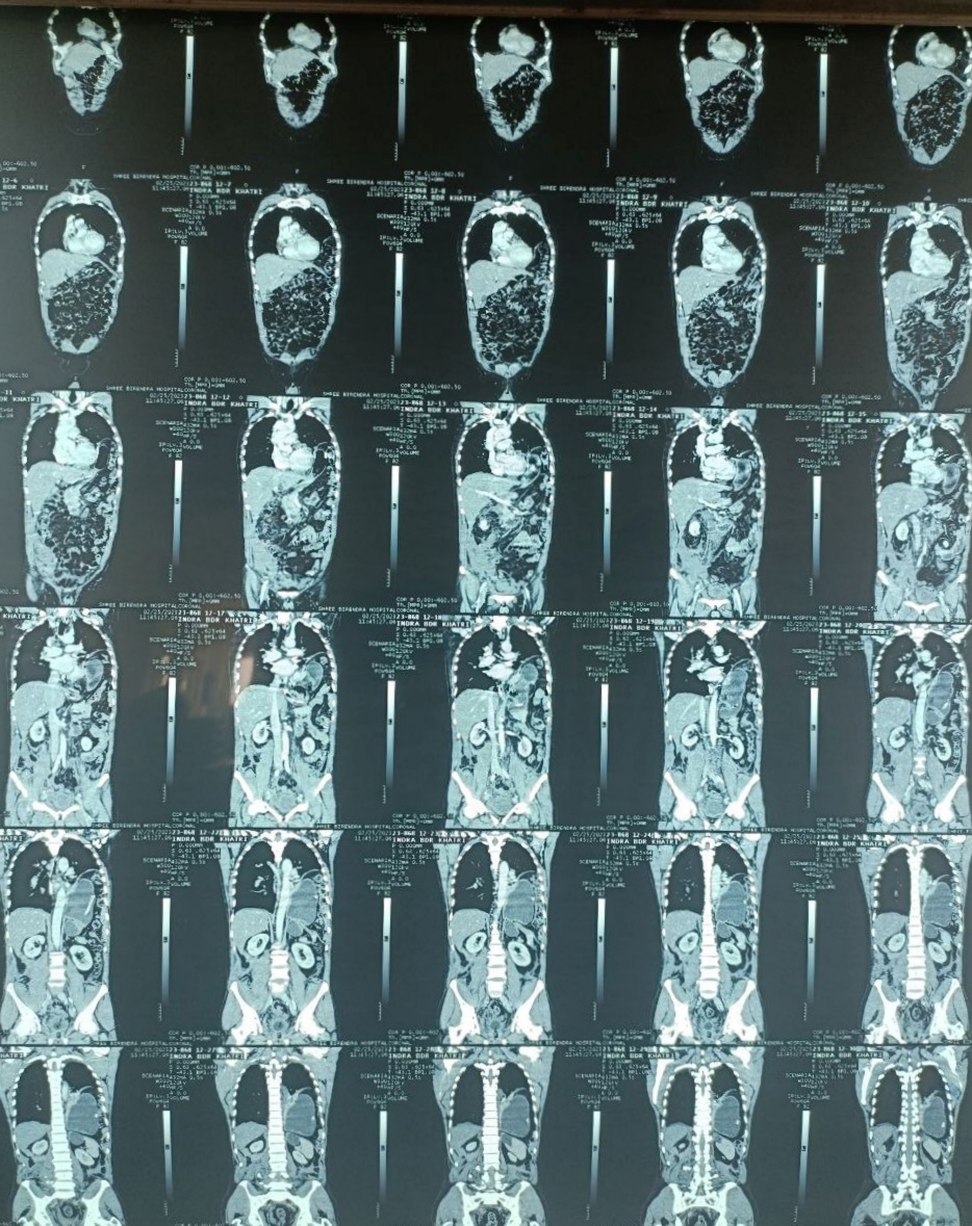
CECT showing defect over the left hemidiaphragm and herniation of fundus and body of stomach, bowel loops and peritoneal fats into left thoracic cavity.

Chest X‐ray of the chest showed elevated left hemidiaphragm (Figure 2). Contrast enhanced computed tomography (CECT) of the chest and abdomen was performed, and the imaging showed fracture at the anterior endplate of LV5 vertebral body; displaced fracture was seen at right transverse process of LV3, LV4, and LV5. Breach in the left hemidiaphragm measuring 5.5 cm with herniation of the fundus and body of stomach, bowel loops, and peritoneal fats into left thoracic cavity with associated passive atelectasis of left lower lobe was appreciated; mild subjacent pleural fluid collection over left side and subtle mediastinal shift to contralateral side were also detected (Figure 3).

With the consideration of physical findings and radiological findings, diagnosis of left diaphragmatic hernia was made from the Surgery side.

### 2.3 Treatment

**FIGURE 4:**
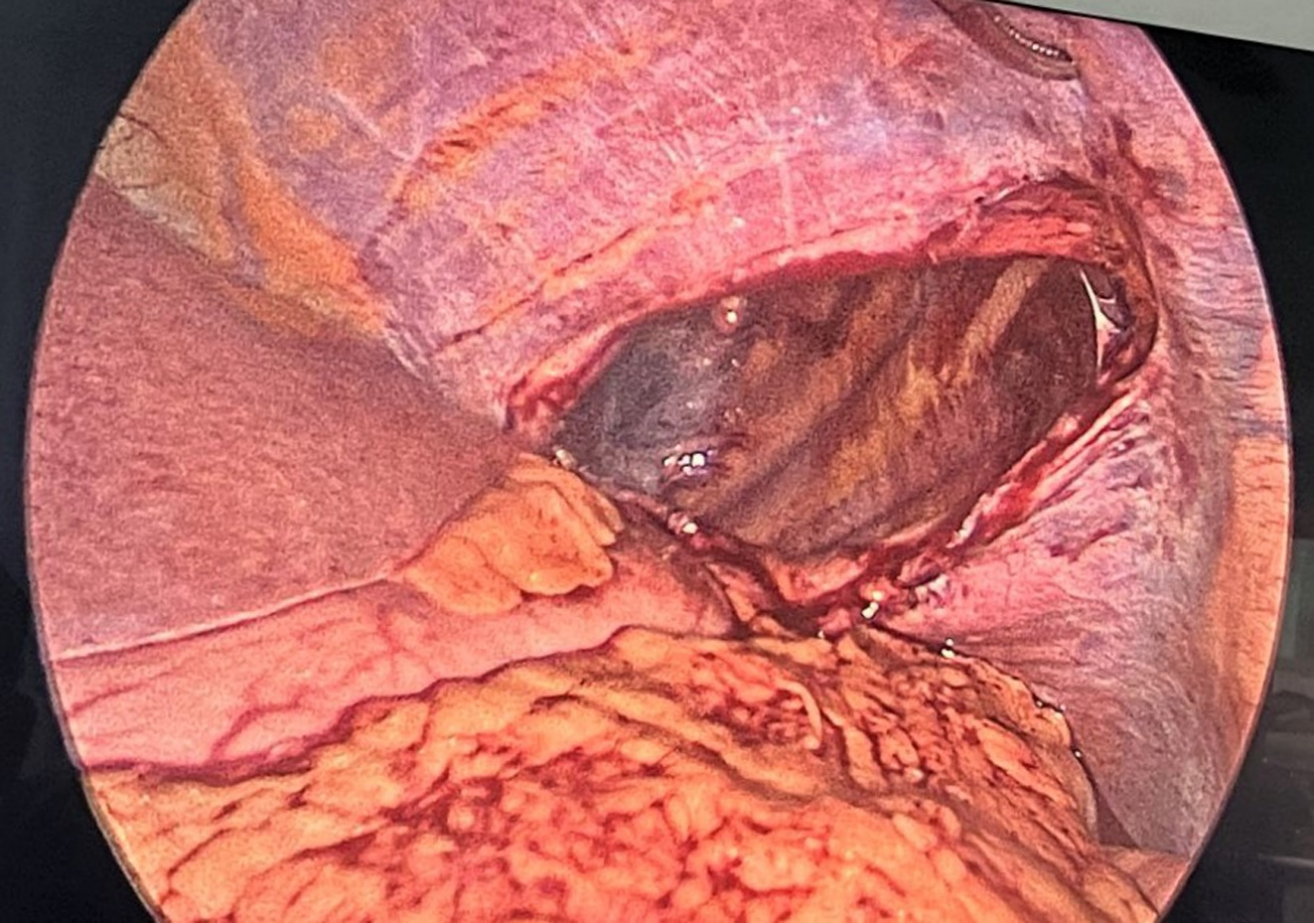
Defect over left hemidiaphragm visualized during the Laparoscopic procedure.

**FIGURE 5:**
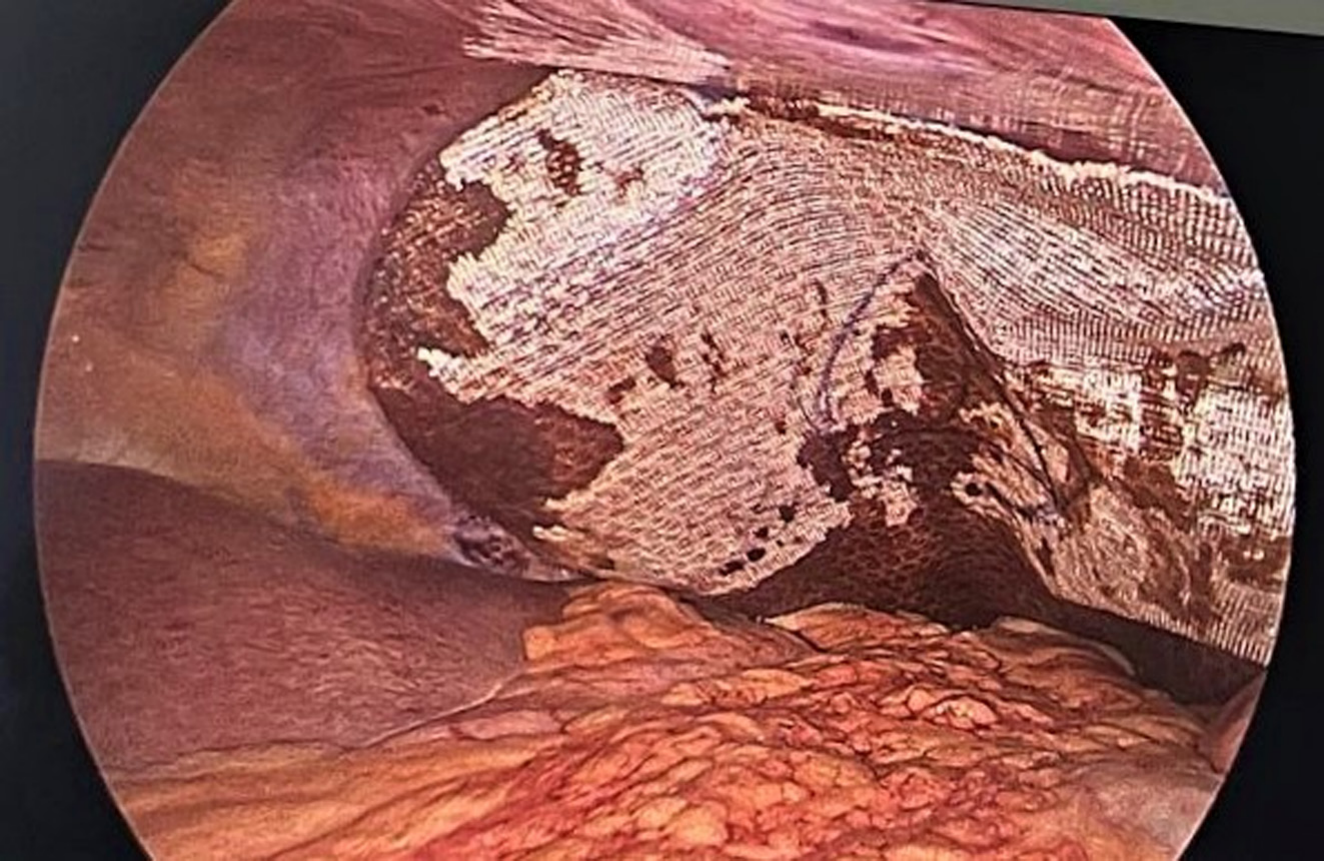
Mesh repair of the defect.

Patient was managed with multidisciplinary approach. Patient was immobilized, and pain management was done. Patient was kept on 2 L/min of oxygen through oxygen mask, and continuous monitoring of the vital parameters was done. Patient was then planned for laparoscopic repair of the diaphragmatic injury from the surgery side. Defect over the left hemidiaphragm was visualized (Figure 4) for which laparoscopic repair was performed. Reduction of herniating contents was performed; then, repair was done by Silk 2–0 suture material; and composite mesh was placed over the repaired region (Figure 5). A chest drain was placed which was removed after a week. After the surgery patient was put on a five‐day course of Cefixime (200 mg, two times a day) and Pantoprazole (40 mg, once a day). Pain management was continued for the patient.

### 2.4 Follow‐up

**FIGURE 6:**
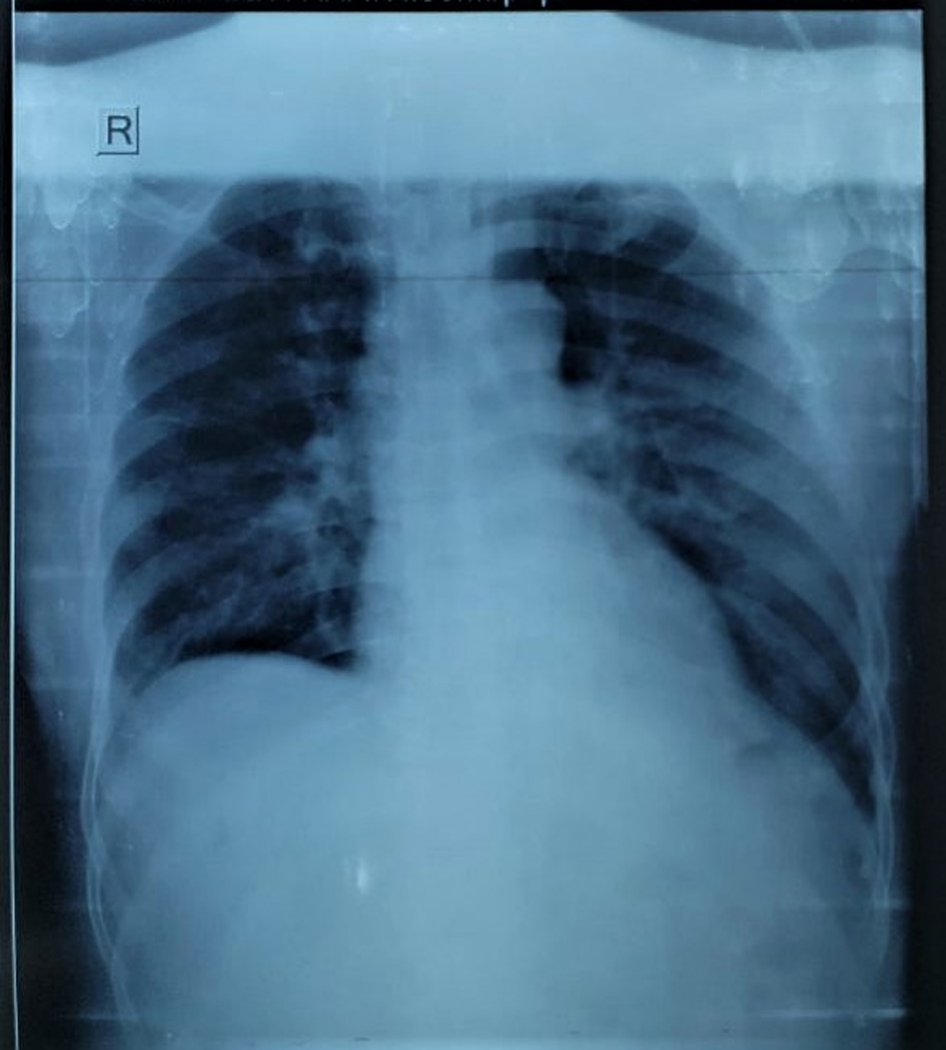
Chest X‐ray on tenth post‐operative day.

After the surgical repair of the patient, patient improved symptomatically and no major post‐operative complications were noted during the hospital stay. Patient's shortness of breath resolved and oxygen supplementation was no longer required. Patient was discharged from the surgery side and handed over to orthopedics department for management of other ailments. Chest X‐ray of the patient at the time of handover is shown in Figure 6.

## 3 DISCUSSION

The incidence of diaphragmatic injury following the blunt thoracoabdominal trauma is 0.8% to 1.6%.^8^ Diaphragmatic rupture is very difficult to diagnose requiring a high index of suspension with patients often being asymptomatic initially but presenting later with higher morbidity and mortality. Likewise, a case of diaphragmatic hernia could present with no symptoms at all or with complaints depending upon the organs herniated into the thoracic cavity and its extent and duration.

The most common organ herniating is the stomach followed by colon, omentum, small intestine, spleen, and liver.^9^ In gastric herniation, patient often complains retching and regurgitation with non‐distended abdomen, while in bowel loop herniation features of intestinal obstruction like upper quadrant pain, pain and heaviness after eating meals, nausea and vomiting with abdominal distension.^3^ The symptomatology of diaphragmatic injury and hernia varies widely depending on the duration. In acute presentation, the complaints are shortness of breath, shoulder pain, and epigastric pain, whereas in late presentation, the symptoms suggestive of intestinal obstructions start to appear.^3^, ^4^, ^10^

Left sided diaphragmatic injury and hernia are more common than right sided diaphragmatic injury and hernia, with bilateral injuries and hernias being the most uncommon one.^11^, ^12^ Diaphragmatic injuries and hernia occur from the weakest point which is the line of embryonic fusion at the posterolateral part of each hemithorax. Also, the left hemidiaphragm is less resistant to the pressure gradient than the right side.^13^ Right hemidiaphragm lies over the liver which prevents the herniation of the abdominal contents even if there is a defect on the diaphragm.^14^

Traumatic diaphragmatic hernia is almost always accompanied by other injuries. The most common injuries noted were splenic injuries, liver injuries, rib fractures, pelvic fractures, and genitourinary injuries,^9^, ^13^, ^15^ none of which was seen in our case. The diagnosis of diaphragmatic rupture is often missed out on chest X‐ray alone, so computed tomography of the chest and abdomen is considered the gold standard investigation for suspected diaphragmatic rupture and hernia.^9^, ^15^

Conventionally, laparotomy has been the surgery of choice but nowadays, even minimally invasive laparoscopic surgeries are successful for diaphragmatic rupture and hernia with the benefits of lesser post‐operative, early return to daily activities and better cosmetic.^10^ The defect can be repaired with the help of sutures with or without the use of mesh as in the case. There are also instances like lacerations of duodenum or pancreas where the laparoscopy is followed by a laparotomy.^10^

## 4 CONCLUSION

Diaphragmatic hernia does not only occur during high velocity impact or penetrating injury, but also can occur when heavy loads impact the torso. Diaphragmatic hernia must be ruled out in a patient with polytrauma with a chest X‐ray at the least. Laparoscopic repair of the rent in the diaphragm provides faster recovery with lesser complications.

## AUTHOR CONTRIBUTIONS

**Oshan Shrestha:** Conceptualization; data curation; methodology; project administration; validation; writing – original draft; writing – review and editing. **Sunil Basukala:** Conceptualization; methodology; project administration; supervision; writing – review and editing. **Sagun Karki:** Data curation; methodology; project administration; validation; writing – original draft; writing – review and editing. **Niranjan Thapa:** Data curation; methodology; project administration; validation; writing – original draft; writing – review and editing. **Niraj Joshi:** Data curation; methodology; project administration; validation; writing – original draft. **Lochan Shrestha:** Project administration; validation; writing – review and editing. **Melina Shrestha:** Data curation; project administration; writing – original draft.

## FUNDING INFORMATION

This article did not receive any grants.

## CONFLICT OF INTEREST STATEMENT

No conflict of interests.

## CONSENT

Written informed consent was obtained from the patient for publication of this case report and accompanying images. A copy of the written consent is available for review by the editor in chief of this journal on request.

## Data Availability

All the findings are present within the manuscript.
